# Supplementary material for: Modeling Human Lung Cells Exposure to Wildfire Uncovers Aberrant lncRNAs Signature
Source: Biomolecules. 2023 Jan 12;13(1):155. doi: 10.3390/biom13010155 (PMC9855943; doi:10.3390/biom13010155)
Supplement: Supplementary file 1 [file biomolecules-13-00155-s001.zip › biomolecules-2106083-supplementary.pdf]

Supplementary Table S1. PAHs concentration (ug/m^3) for the two different fuel smokes

Compounds	Smoke A			Smoke B		
	Total	Particle	Gas	Total	Particle	Gas
1-ethyl-2-methylnaphthalene	0.090		0.090	0.219		0.219
1-methylfluorene	2.108	2.020	0.088	8.299	8.160	0.139
1-methylnaphthalene	38.27	0.323	37.94	16.22	0.250	15.97
1,2-dimethylnaphthalene	0.783		0.783	1.004	0.211	0.793
1,3+1,6+1,7-dimethylnaphth	10.45	3.446	7.007	5.563	0.735	4.828
1,4,5-trimethylnaphthalene	15.43	12.74	2.683	1.336	0.784	0.552
1,4+1,5+2,3-dimethylnaphth				1.021	0.180	0.842
1,7-dimethylphenanthrene				0.480	0.480	
1,8-dimethylnaphthalene				0.137		0.137
2-methylbiphenyl	0.445	0.375	0.070	0.227	0.182	0.045
2-methylnaphthalene	38.05	0.396	37.65	22.14	0.542	21.60
2,3,5+i-trimethylnaphthalene	1.437	1.190	0.247	0.701	0.307	0.394
2,4,5-trimethylnaphthalene	16.83	13.58	3.245	3.208	1.653	1.555
2,6+2,7-dimethylnaphthalene	1.775	0.489	1.286	3.331	1.673	1.657
3-methylchrysene				0.096	0.096	
3,6-dimethylphenanthrene				0.045	0.045	
4-methylpyrene	0.504	0.504		0.523	0.522	0.001
4,5-methylenephenanthrene	0.963	0.962	0.002	0.562	0.555	0.008
7-methylbenz[a]anthracene	0.809	0.809				
a-methylfluorene	2.321	2.227	0.094	1.986	1.827	0.159
acenaphthene	1.436	0.945	0.490	0.721	0.224	0.497
anthanthrene	0.156	0.156		0.185	0.185	
anthrone	14.12	14.12				
b-dimethylphenanthrene	0.442	0.442		0.190	0.190	
b-methylfluorene				0.057		0.057
b-trimethylnaphthalene	1.810	1.567	0.243	0.506	0.238	0.268
benzo[a]fluoranthene	0.227	0.227		0.361	0.361	
benzo[a]fluorene	0.574	0.574		0.341	0.341	
benzo[a]pyrene	0.533	0.533		0.664	0.657	0.006
benzo[b]chrysene	0.084	0.084		0.098	0.098	
benzo[b]fluorene	0.255	0.255		0.137	0.137	
benzo[c]phenanthrene	0.334	0.334		0.324	0.324	
benzo[e]pyrene	0.264	0.264		0.394	0.394	
benzo[ghi]perylene	0.352	0.352		0.540	0.540	
benzo[j]fluoranthene	0.466	0.466		0.681	0.681	
benzo[k]fluoranthene	0.366	0.366		0.393	0.393	
c-mepy/mefl	0.556	0.556		0.210	0.210	
c-trimethylnaphthalene	3.272	2.188	1.085	0.961	0.335	0.626
coronene	0.107	0.107		0.155	0.155	

cyclopenta[c,d]pyrene				0.829	0.829	
d-mepy/mefl	1.240	1.240		0.306	0.306	
dibenzo[a,e]pyrene	0.084	0.084		0.095	0.095	
dibenzo[a,h]anthracene	0.110	0.110		0.122	0.122	
dibenzo[a,h]pyrene	0.273	0.273		0.100	0.100	
dibenzo[a,i]pyrene	0.148	0.148				
dibenzo[a,j]anthracene	0.048	0.048		0.076	0.076	
dibenzo[a,l]pyrene	0.075	0.075		0.104	0.104	
dibenzo[a,c]anthracene				0.031	0.031	
dibenzo[b,k]pyrene				0.109	0.109	
dibenzofuran	3.368	2.282	1.085	7.346	4.947	2.399
e-trimethylnaphthalene	2.048	1.776	0.273	0.762	0.357	0.404
f-trimethylnaphthalene	1.179	0.838	0.341	0.765	0.314	0.450
fluorene	3.978	3.270	0.708	9.284	8.266	1.017
indeno[123-cd]fluoranthene				0.064	0.064	
j-trimethylnaphthalene	1.003	0.751	0.252	0.337	0.159	0.178
naphthalene	119.7	1.263	118.4	73.78	0.129	73.65
perylene	0.080	0.080		0.121	0.121	
phenanthrene	6.518	6.315	0.204	5.155	4.444	0.711
picene	0.101	0.101		0.118	0.118	
triphenylene				0.166	0.166	
xanthone	2.206	2.140	0.066	0.559	0.559	
Total	297.8	83.40	214.38	174.3	45.08	129.2
